# The Project Collection Food, Nutrition and Health, with a Focus on Eating Together

**DOI:** 10.3390/ijerph18041572

**Published:** 2021-02-07

**Authors:** Agneta Yngve, Nicklas Neuman, Irja Haapala, Henrik Scander

**Affiliations:** 1 Department of Food Studies, Nutrition and Dietetics, Uppsala University, 751 22 Uppsala, Sweden; nicklas.neuman@ikv.uu.se; 2 School of Health Sciences, Örebro University, 702 81 Örebro, Sweden; 3 School of Social and Political Sciences, The University of Melbourne, Parkville VIC 3010, Australia; irja.haapala@unimelb.edu.au; 4 School of Hospitality, Culinary Arts and Meal Science, Örebro University, 712 02 Grythyttan, Sweden; henrik.scander@oru.se

## 1. Introduction

Papers in this project collection arise from international networking on interdisciplinary research into commensality. The network was initiated in 2014 by funding provided by the Centre for Integrated Research on Culture and Society (CIRCUS), Uppsala University, for interdisciplinary networking within the university with the aim of expanding to international collaboration with external funding. In 2020, the network produced this series of papers and looks forward to further collaboration which may involve joint seminars and courses related to the topic. We remain happy to have discovered many new colleagues with similar research interests, using perhaps different definitions and methods of approaching the topic, but nevertheless sharing the devotion to the same phenomenon: commensality.

Eating together with other people (engaging in commensality) is a central manifestation of human sociality, including the formation of social cohesion and indication of social distinctions [1,2,3]. Some examples of the wide array of empirical applications of the commensality concept include the role of meals in political high-level meetings [4], intersections of class and gender at dinner parties in the United States of America [5], childhood interaction around school lunch in Denmark [6], meals among people with intellectual disabilities living in the community in Sweden [7], food in the lives of Scandinavian men of different ages [8,9] and meal breaks for the performance of fire fighters [10].

The concept has also been used to analyze cultural variations in festive eating [11] and Jewish cultural identity [12]. In relation to the latter, theologians have studied commensality from a mostly ritualistic, religious point of view, but also as a symbol of identity [13,14] and obviously as an important part of sociality. Moreover, commensality has been a central concept in contemporary contributions to the sociology of food, where commensality is studied as a phenomenon that manifests both social change and stability. Examples of this are the everyday eating in Nordic countries [15], eating out in England [16], time-use in Belgium [17] and the Netherlands [18], meal synchronization in Santiago and Paris [19], meal arrangements among Britons [20] and the habits and orders of everyday life among Anglo-French couples [20,21].

In current nutrition research and policy, commensality has been emphasized as having a role to play in supporting optimal nutritional health and food enjoyment, especially in later life [22,23,24], a subject touched upon by some of the papers in this issue. In the decision by UNESCO to acknowledge the Mediterranean diet (MD) as an important intangible heritage of humanity, the following statement is included: “The Mediterranean Diet emphasizes values of hospitality, neighborliness, intercultural dialogue and creativity and a way of life guided by respect for diversity” [25]. In a consensus paper on the MD, described in more detail by Xavier Medina in this issue, commensality is again mentioned as an important part of the sociocultural historical heritage [26]. While some national nutritional recommendations, such as the Nordic Nutrition Recommendations [27], have not mentioned the role of commensality, “eating together” has made its way into official dietary advice in countries such as Canada [28], Japan [29], and Brazil [30].

Current discussion around commensality within households tends to focus on the location where people choose to eat, for example, in front of the TV or at the table, and whether the change of location leads to nutritionally unfavorable food choices or not [31]. Food choice in regards to eating together or alone has not been extensively studied, but some publications have pointed to a risk of less healthy meals among older adults who have become widowed [24] and thus may eat alone, and several to associations between family-meal participation frequency and food intake among children [32]. There are also examples of evidence to the contrary, with less healthy choices of food and drink sometimes occurring when eating together, for example during weekends, enjoying extra meals for their aesthetic pleasure, or having meals just to keep company, as indicated by the latest National Food Survey in Sweden [33], or during workplace breaks [34,35]. However, a later review points at the importance or potential importance of having regular commensal meals [36]. In other studies, not only eating together, but also cooking together has been emphasized as very important [37,38]. So far, research has not clearly identified a causal relationship between eating together and food choice. 

## 2. Scope of the Project Collection

Viewpoints on the above issues are discussed in several of the contributions to this project collection. As exemplified above, commensality can be studied in relation to a number of current developments from different societies and time periods. Current issues include, for example, dining area planning in workplaces, homes, restaurants, and institutions. New mealtime patterns at work and at home, both alone and with others, shape contemporary commensality and present new possibilities for research. We need to study who eats with whom, in what context, and for how long, both in and outside of single households and families, and in settings where different generations can eat together, in private and in public. Restrictive diets and food allergies have become better known and accepted, and where they put a strain on commensality, we need to understand such constraints. Innovations in the restaurant industry, media, and communications also widen the possibilities for commensality, as do new food trends among customers. Moreover, the implications of this improved understanding for methodological development, theory, business, and public policy need to be elucidated in order to promote wellbeing in individuals as well as in organizations. The impact of sharing a meal with someone you would not have chosen yourself is another important research topic. This could have an impact on conviviality as well as on nutritional health; for example, for people in aged care, work, or school settings. It may also be important to consider in the restaurant business, where formal occasions and ceremonial meals may involve prearranged seating which could have an effect, positive or negative, on the conviviality of eating together. 

## 3. Current Effects on Commensality

In the light of the current coronavirus disease 2019 (COVID-19) pandemic, we can identify a number of important future research topics related to the ways the pandemic may have changed eating patterns in general and commensal patterns in particular. These include: To what extent have people’s commensal patterns changed due to the government-imposed restrictions on restaurant operating hours and the number of guests per table?; To what extent have these affected the use of take-away food and cooking at home?; To what extent have people experienced the feeling of being “all dressed up but having nowhere to go” or having food in the house but lacking opportunities to share it with loved ones categorized as part of the COVID-19 vulnerable groups?. In the WHO dietary guidelines for quarantine during COVID-19, eating together is mentioned as one of the recommendations [39], but opportunities for commensality may have become limited and mediated by computer technology to take place at a distance. 

## 4. The Joy and Importance of Networking

Through networking, common views across scientific boundaries can be reached and new research and interventions be discussed and developed. The papers in this collection arose from collaboration, firstly within Uppsala University, Sweden, then spread nationally to: the Swedish University of Agricultural Sciences, Future Foods; Linnaeus University; University of Gävle; Örebro university; University of Lund; and internationally, to: University of Porto, Portugal; University of Barcelona, Spain; Institute Paul Bocuse, Lyon, France; Université Lumière-Lyon 2, France; te University of Melbourne, Australia; and Flinders University, Adelaide, Australia. By working and eating together, and enjoying each other’s company, we hope that we have contributed to the spirit of commensality both conceptually and empirically. 
